# Trans-heteroclinic bifurcation: a novel type of catastrophic shift

**DOI:** 10.1098/rsos.171304

**Published:** 2018-01-24

**Authors:** Josep Sardanyés, Regina Martínez, Carles Simó

**Affiliations:** 1 Centre de Recerca Matemàtica, Campus de Bellaterra, Edifici C, 08193 Bellaterra, Barcelona, Spain; 2 Barcelona Graduate School of Mathematics (BGSMath) Campus de Bellaterra, Edifici C, 08193 Bellaterra, Barcelona, Spain; 3 Departament de Matemàtiques, Edifici C, Universitat Autònoma de Barcelona 08193 Bellaterra, , Spain; 4 Departament de Matemàtiques i Informàtica (Universitat de Barcelona), Gran Via de les Corts Catalanes 585, 08007 Barcelona, Spain

**Keywords:** global bifurcations, nonlinear dynamics, quasi-species equations, catastrophic shifts, trans-heteroclinic bifurcation, applied mathematics

## Abstract

Global and local bifurcations are extremely important since they govern the transitions between different qualitative regimes in dynamical systems. These transitions or tipping points, which are ubiquitous in nature, can be smooth or catastrophic. Smooth transitions involve a continuous change in the steady state of the system until the bifurcation value is crossed, giving place to a second-order phase transition. Catastrophic transitions involve a discontinuity of the steady state at the bifurcation value, giving place to first-order phase transitions. Examples of catastrophic shifts can be found in ecosystems, climate, economic or social systems. Here we report a new type of global bifurcation responsible for a catastrophic shift. This bifurcation, identified in a family of quasi-species equations and named as *trans-heteroclinic bifurcation*, involves an exchange of stability between two distant and heteroclinically connected fixed points. Since the two fixed points interchange the stability without colliding, a catastrophic shift takes place. We provide an exhaustive description of this new bifurcation, also detailing the structure of the replication–mutation matrix of the quasi-species equation giving place to this bifurcation. A perturbation analysis is provided around the bifurcation value. At this value the heteroclinic connection is replaced by a line of fixed points in the quasi-species model. But it is shown that, if the replication–mutation matrix satisfies suitable conditions, then, under a small perturbation, the exchange of heteroclinic connections is preserved, except on a tiny range around the bifurcation value whose size is of the order of magnitude of the perturbation. The results presented here can help to understand better novel mechanisms behind catastrophic shifts and contribute to a finer identification of such transitions in theoretical models in evolutionary biology and other dynamical systems.

## 1. Introduction

Bifurcations, both local and global, govern transitions between qualitatively different states in dynamical systems. Bifurcations have been experimentally reported in physics [1–4], chemistry [5,6] and biology [7,8]. Bifurcations in biological dynamical systems can govern extinction processes and population collapses [7,9–14]. Such extinctions can be smooth or catastrophic when one or more bifurcation parameters are tuned.

Smooth transitions, found in some natural and social systems, cause soft changes between active and quiescent states, with a more easily reversed progressive deterioration [15]. These transitions can occur in chemical reactions, epidemic spreading, fixation of alleles in population genetics and computer virus propagation [16–20]. Typically, smooth transitions are governed by transcritical bifurcations, although other types of bifurcations such as pitchfork [21] or saddle–node bifurcations (whenever the node and the saddle collide at a point located at the boundary of the admissible domain, see e.g. [22]) can give place to continuous population declines until extinction is achieved. The transcritical bifurcation typically involves the collision of two fixed points and an exchange of stability between them [23].

Opposite to smooth shifts, other bifurcations can cause the so-called catastrophic transitions [9,10]. For instance saddle–node bifurcations (whenever the saddle and the node collide at the interior of the admissible domain and the orbits that where attracted by the node jump to a different, distant, attractor). This bifurcation, typical of catalytic systems [24,25], has been recently mapped in laboratory experiments with yeast [7]. Saddle–node bifurcations are also found in food-chain mathematical models [26] or in host–parasite theoretical models [27]. Also, global bifurcations such as saddle–nodes of periodic orbits [28], homoclinic bifurcations [29,30], or the so-called chaotic crises [31–33] (which are mainly created by tangential heteroclinic events [34]) can give place to discontinuous shifts. Catastrophic shifts are of extreme importance in nature since they involve a radical change in the steady state of a system once the bifurcation threshold is crossed [13,14]. Sudden collapses can occur in socio-ecological systems [35–37] as well as in ecosystems. For instance, lakes, fisheries, coral reefs or savannahs can suffer abrupt collapses due to small changes in environmental conditions [30,38–43]. Once such collapse occurs, recovery can be extremely problematic [15,30].

In this paper, we carry out a general study of a new type of global bifurcation recently identified in the quasi-species equation [44]. This bifurcation, named as *trans-heteroclinic*, involves a catastrophic shift. Identifying the mechanisms responsible for catastrophic regime shifts and distinguishing them from their smoother counterparts constitutes a timely subject with enormous important applications. We start by introducing the mathematical model where this bifurcation was found to provide some background for what is followed. Next, we provide a description of this new type of transition involving a discontinuous shift. For values of the parameter below the critical one there exists a heteroclinic connection from a point, say *Q*_1_, to a distant point, say *Q*_2_. After crossing the critical value the heteroclinic connection is recovered, but the stability roles of *Q*_1_ and *Q*_2_ are exchanged. We provide the conditions (within the replication–mutation matrix) needed for this bifurcation to take place in the general quasi-species replicator model. Then, we develop a perturbation analysis around the bifurcation value.

## 2. Results

### 2.1. Background

In the following lines we introduce the mathematical model that displays a trans-heteroclinic bifurcation. This model was obtained from the well-known Eigen’s quasi-species equation [45] (see electronic supplementary material, §1), and it considers a heterogeneous pool of tumour cell phenotypes competing with healthy cells [44]. Assuming a constant population, Eigen’s model can be represented with the general equation:

2.1 $$\dot{\text{x}}=A\text{x}-\Phi (\text{x})\text{x},$$

where $\text{x}=(x_{1},\ldots ,x_{n})\in R^{n}$ is the population vector with all components non-negative and normalized initially by **u**^*T*^**x**(0)=1 where ${\text{u}}^{T}=(1,1,\ldots ,1)\in R^{n}$. Equation (2.1) can be further generalized to the dynamic equations describing self-replicating species in a chemostat. *A* is an *n*×*n* matrix with all entries non-negative and corresponds, for Eigen’s model, to the replication–mutation matrix, which includes all the terms tied to the replication and mutation rates of the sequences *x*_*i*=1…*n*_. The entries of matrix *A*, which determine the shape of the fitness landscape and how the quasi-species move across this landscape, can also be represented as *a*_*ij*_=*f*_*i*_*Q*_*ji*_, being *f*_*i*_ the fitness of sequence *i* (i.e. replication rates) and *Q*_*ji*_ the mutation matrix [46]. *Φ* is the so-called outflow term, given by a linear function of **x** that introduces competition and keeps the population constant so that **u**^*T*^**x**=1 is preserved for all *t*. That is $\Phi (\text{x})={\text{u}}^{T}A\text{x}=\sum_{j=1}^{n}(\sum_{i=1}^{n}a_{i,j})x_{j}$.

We remark that an eigenvector **v** of *A* with non-negative components and normalized such that **u**^T^**v**=1, determines a fixed point of the quasi-species equation. The corresponding eigenvalue coincides with *Φ*(**v**). It is well known that explicit solutions can be obtained for Eigen’s quasi-species model [47,48] by introducing a vector $\text{y}\in R^{n}$ defined as

$$\text{y}=\exp(f(t))\text{x},\;f(t):=\int_{0}^{t}\Phi (\text{x}(\tau ))\;d\tau ,$$

where the integration is done along a solution **x**(*τ*). Then the equation for **y** becomes $\dot{\text{y}}=A\text{y}$, easy to solve, and **x**(*t*) is recovered as **y**(*t*)/**u**^*T*^**y**(*t*).

**Figure 1.**
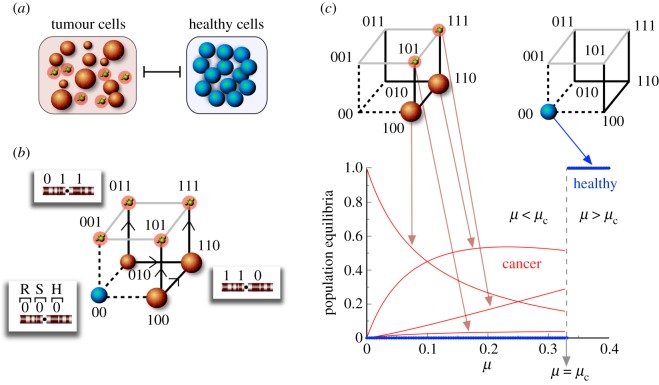
The trans-heteroclinic bifurcation in a cancer quasi-species model. (*a*) Schematic diagram of the model, in which different tumour phenotypes compete with healthy cells (see [44]). (*b*) Structure of the sequence space for the cancer phenotypic model. Here tumour phenotypes include cells with anomalies in replication-related genes (proto-oncogenes and tumour suppressor genes, compartment *R*); anomalies in genes preserving genomic stability (compartment *S*); and anomalies in housekeeping genes (compartment *H*). Alterations in these compartments are assumed to define different cancerous phenotypes. (*c*) Catastrophic transition governed by the trans-heteroclinic bifurcation when increasing the mutation rate of tumour cells above a critical threshold *μ*_c_ [44]. This bifurcation separates two different scenarios: (i) persistence of tumour cells and vanishing of the healthy ones when *μ*<*μ*_c_ and (ii) extinction of tumour cells and dominance of healthy cells when *μ*>*μ*_c_.

Equation (2.1) provides a general model for investigating replication–mutation dynamics. This equation can be adapted to investigate the dynamics of different systems such as the one displayed in figure 1, which is here introduced as a model example of the trans-heteroclinic bifurcation [44]. Specifically, this model has been recently used to explore the population dynamics of healthy cells (labelled *x*_0_) competing with tumour cells phenotypes (*x*_1…7_). These cells can carry mutations or present anomalies in genes involved in cell growth (compartment *R* in figure 1*b*, i.e. proto-oncogenes and tumour suppressor genes), in genes keeping genomic stability (compartment *S*), and in housekeeping genes (compartment *H*, see [44] for details). The model parameters are given by the replication rate of cells *r*>0; by the increase in proliferation of tumour cells due to anomalies in compartment *R*, given by *δ*_*r*_>0; by the tumour cells mutation rates 0<*μ*<1; and by the increase in mutation rates (*δ*_*μ*_) of tumour cells with anomalies in compartment *S*, with 0<*δ*_*μ*_<1−*μ*. As was done in [44], we introduce $\hat{r}=r+\delta_{r},$ $\hat{\mu }=\mu +\delta_{\mu }$, $\alpha_{1}=\hat{\mu }r/2$ and $\alpha_{2}=\mu \hat{r}/2$. According to the previous terms, the dynamical system introduced in [44] and built from equation (2.1) can be written in a compact form as

2.2 $$\begin{array}{rl} {\dot{x}}_{0} & =x_{0}(r-\Phi ),\\ {\dot{x}}_{2} & =x_{2}((1-\hat{\mu })r-\Phi ),\\ {\dot{x}}_{4} & =x_{4}((1-\mu )\hat{r}-\Phi ),\\ {\dot{x}}_{6} & =x_{6}((1-\hat{\mu })\hat{r}-\Phi )+\alpha_{1}x_{2}+\alpha_{2}x_{4},\\ {\dot{x}}_{1} & =-x_{1}\Phi ,\\ {\dot{x}}_{3} & =\alpha_{1}x_{2}-x_{3}\Phi ,\\ {\dot{x}}_{5} & =\alpha_{2}x_{4}-x_{5}\Phi ,\\ \;\text{and}\;\;{\dot{x}}_{7} & =\hat{\mu }\hat{r}x_{6}-x_{7}\Phi , \end{array}\}$$

where $\Phi =r(x_{0}+x_{2})+\hat{r}(x_{4}+x_{6})$. The model above displays a threshold behaviour at a critical mutation rate, *μ*_c_=*δ*_*r*_/(*r*+*δ*_*r*_), resulting in a discontinuous or jump transition. This catastrophic shift, caused by a trans-heteroclinic bifurcation, is displayed in figure 1*c*, where the equilibrium populations of cells are plotted against *μ*. When *μ*<*μ*_c_ the whole population is dominated by tumour cells. However, at *μ*=*μ*_c_ a discontinuous transition occurs, resulting in an abrupt and catastrophic extinction of the tumour cells and a dominance of healthy cells (that persists for *μ*>*μ*_c_).

**Figure 2.**
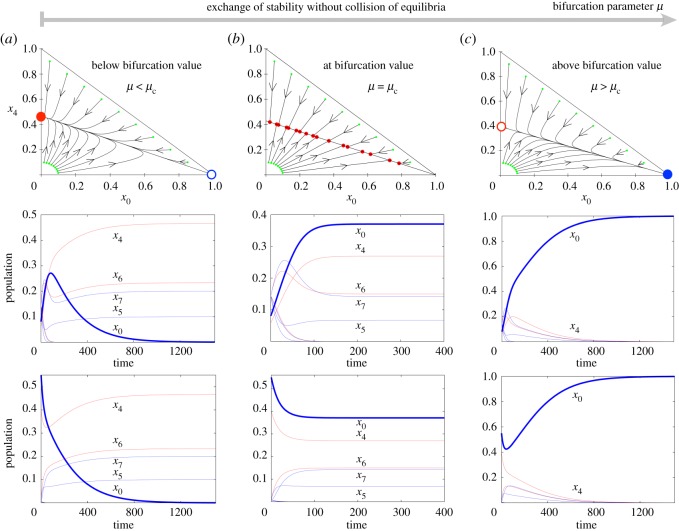
Topological changes associated to the trans-heteroclinic bifurcation. Phase portraits for system equations (2.2) with *r*=0.1, *δ*_*r*_=0.05 and *δ*_*μ*_=0.05 displayed in the projection (*x*_0_,*x*_4_). (*a*) Orbits attracted by the fixed point $P_{3}^{*}$ (red solid circle, which is stable with $\mu_{c}>\mu =\frac{3}{10}$) with *x*_0_=0 (see [44] for notation) before the trans-heteroclinic bifurcation. (*b*) Dynamics at the bifurcation value $\mu =\mu_{c}=\frac{1}{3}$, for which the final state depends on the initial conditions (the small red dots indicate the asymptotic population state). (*c*) Phase portrait above *μ*_c_, with $\mu_{c}<\mu =\frac{11}{30}$. Here the orbits achieve the fixed point $P_{2}^{*}$ with *x*_0_=1 (see [44]), which is globally asymptotically stable (blue solid circle). Notice that in all panels, the trajectories are attracted by the heteroclinic connection. Stable (unstable) fixed points are indicated with solid (open) circles. On each panel one has taken nine initial conditions on top and below the line joining $P_{2}^{*}$ and $P_{3}^{*}$. The time series in the second (resp. third) row correspond to initial data taken below (resp. on top) of the line joining $P_{2}^{*}$ and $P_{3}^{*}$ in the related phase portrait. Here the dynamics of healthy (*x*_0_ variable, thick blue line) and tumour cells populations (thin lines in red, except for lethal phenotypes, indicated with blue thin lines) are represented. All of the variables are plotted although some of them appear overlapped or go rapidly to extinction.

The topological changes of equation (2.2) are represented in figure 2, below (*a*), at (*b*), and above (*c*) the bifurcation. When *μ*<*μ*_c_, the fixed point (1,0,…,0) is unstable and the flow achieves a globally asymptotically stable fixed point that involves the dominance of tumour cell phenotypes (red circle in figure 2*a*). Just at the bifurcation value *μ*=*μ*_c_ the heteroclinic connection between the two alternative states (dominance of tumour cells versus dominance of healthy cells) is a line of fixed points, and the asymptotic states largely depend on the initial conditions (figure 2*b*). Finally, once *μ*>*μ*_c_, the fixed point (1,0,…,0) becomes a global attractor (blue circle in figure 2*c*) due to the stability exchange between the two equilibria. Figure 2 also displays time series associated to each one of the different scenarios found below, at and above the bifurcation.

We note that the same catastrophic shift is found in a quasi-species mathematical model describing the dynamics of the so-called survival-of-the-flattest effect (see [49]). The trans-heteroclinic bifurcation for this dynamical system is discussed in electronic supplementary material, §2.

Next, we provide a description of the trans-heteroclinic bifurcation and the structure of the replication–mutation matrix giving place to this type of catastrophic shift.

### 2.2. Structure of the replication–mutation matrix to find the trans-heteroclinic bifurcation

We consider the general quasi-species model given by equation (2.1). According to the Perron–Frobenius theorem the matrix *A* has a positive dominant eigenvalue, λ_1_, such that all the other eigenvalues, λ_*j*_,*j*>1, satisfy λ_1_≥*Re*(λ_*j*_). Furthermore, if the matrix *A* is irreducible, that is, there does not exist a permutation matrix *P* so that $PAP^{T}=\left(\begin{array}{cc} B & 0\\ C & D \end{array}\right)$, where *B* and *D* are square *k*×*k* and (*n*−*k*)×(*n*−*k*) matrices, respectively, with 0<*k*<*n*, then λ_1_ has multiplicity 1 and there exists a non-trivial eigenvector, say **v**_1_, with eigenvalue λ_1_, which has all the components positive. Hence, if *A* is irreducible and we consider an initial vector **y**(0)=**x**(0) with non-zero component in **v**_1_, the solution tends to the fixed point associated to this eigenvector, and it is an attracting fixed point of the system.

We would like to have a line of fixed points. So, we should assume that *A* is reducible, with a block triangular structure as mentioned before. The matrix, after a suitable permutation, can have a 2×2 block structure or a higher number of blocks. Then it is possible to have a dominant eigenvalue λ_1_ with algebraic multiplicity equal to 2. But if in the associated subspace the matrix has a non-diagonal Jordan structure (that is, the geometrical multiplicity is 1) there appear terms of the form $t\exp(\lambda_{1}t)$ in the solution, which are the dominant ones (assuming that the coefficient is non-zero). In this case, one has again a single attracting fixed point.

The matrix *A* can depend continuously on several parameters such as replication and mutation rates. For concreteness we assume all of them fixed except one of them, say *μ*, and we shall consider *A*(*μ*). Then the eigenvalues depend on *μ* in a continuous way.

The next proposition gives sufficient conditions so that the quasi-species equation has a trans-heteroclinic bifurcation at some critical value *μ*=*μ*_c_.

**Proposition 2.1**

*Assume that the matrix* *A*(*μ*), *with all entries non-negative, depends on a parameter* *μ*. *Assume that there exists a critical value* *μ*_c_ *such that the following conditions hold*:

(1) *For* *μ*=*μ*_c_ *the matrix* *A*(*μ*) *has a dominant eigenvalue with algebraic and geometric multiplicity equal to 2*
  $$\lambda_{1}(\mu_{c})=\lambda_{2}(\mu_{c})>\text{Re}(\lambda_{j}(\mu_{c})),\;j=3,\ldots ,n$$
  *and two independent eigenvectors* ***v***_1_(*μ*_c_),***v***_2_(*μ*_c_) *for the eigenvalue* λ_1_(*μ*_c_)*, with non-negative components.*
(2) *For* *μ* *close to* *μ*_c_
  $$\lambda_{1}(\mu )>\lambda_{2}(\mu )>\text{Re}(\lambda_{j}(\mu )),\;j=3,\ldots ,n,\;\text{if}\;\mu >\mu_{c}$$
  *and*
  $$\lambda_{2}(\mu )>\lambda_{1}(\mu )>\text{Re}(\lambda_{j}(\mu )),\;j=3,\ldots ,n,\;\text{if}\;\mu <\mu_{c}.$$

*Then, for any* *μ* *in a small neighbourhood of* *μ*_c_, *there is an attracting invariant line* *L*(*μ*) *such that for* *μ*=*μ*_c_ *all points in the line are fixed, and for* *μ*≠*μ*_c_*, there are in* *L*(*μ*) *two fixed points* *Q*_1_(*μ*),(*μ*)*, and a heteroclinic connection, from* *Q*_2_(*μ*) *to* *Q*_1_(*μ*) *if* *μ*>*μ*_c_ *and from* *Q*_1_(*μ*) *to* *Q*_2_(*μ*) *if* *μ*<*μ*_c_. *In this case we say that a trans-heteroclinic bifurcation appears for the critical value* *μ*_c_.

**Proof.**

From now on all the eigenvectors **v** of *A* are assumed to be normalized, that is, **u**^T^**v**=1.

Let **v**_1_(*μ*),**v**_2_(*μ*) be eigenvectors for λ_1_(*μ*),λ_2_(*μ*), respectively. The line defined by **x**=*c***v**_1_(*μ*)+(1−*c*)**v**_2_(*μ*), c∈R is invariant. Notice that if we take initial conditions in that line, that is, **x**(0)=*c***v**_1_(*μ*)+(1−*c*)**v**_2_(*μ*) then the solution is given by

2.3 $$\text{x}(t)=\frac{c\;e^{\lambda_{1}t}{\text{v}}_{1}(\mu )+(1-c)\;e^{\lambda_{2}t}{\text{v}}_{2}(\mu )}{c\;e^{\lambda_{1}t}+(1-c)\;e^{\lambda_{2}t}}.$$

Clearly it belongs to the line for any real *t*. Furthermore, if **v**_1_(*μ*),**v**_2_(*μ*) have non-negative components and *c*∈[0,1], then **x**(*t*) has non-negative components for all *t*.

Consider first the case *μ*=*μ*_c_. For any *c* real, *c***v**_1_(*μ*_c_)+(1−*c*)**v**_2_(*μ*_c_) is an eigenvector of *A* for the eigenvalue λ_1_:=λ_1_(*μ*_c_)=λ_2_(*μ*_c_), giving rise to a fixed point. Then we obtain a line of fixed points. Moreover, for any initial condition **x**(0) with non-zero component in **v**_1_ or **v**_2_, the solution can be written as

$$\text{x}(t)=\frac{c_{1}{\text{v}}_{1}(\mu_{c})+c_{2}{\text{v}}_{2}(\mu_{c})+e^{-\lambda_{1}t}\text{Y}(t)}{c_{1}+c_{2}+e^{-\lambda_{1}t}{\text{u}}^{T}\text{Y}(t)},$$

where **Y**(*t*) is a linear combination of functions of the form *e*^λ_*j*_*t*^, for *j*>2 (eventually multiplied by a polynomial in *t* if λ_*j*_ is a multiple eigenvalue) and so, *e*^−λ_1_*t*^**Y**(*t*) tends to **0** as *t* goes to ∞. Therefore, if $c_{1}^{2}+c_{2}^{2}>0$, the solution tends to a fixed point in the line before.

Assume *μ* near *μ*_c_. The invariant line contains two fixed points *Q*_1_(*μ*) and *Q*_2_(*μ*) associated to **v**_1_(*μ*) and **v**_2_(*μ*) respectively. Given an initial condition on the line **x**=*c***v**_1_(*μ*)+(1−*c*)**v**_2_(*μ*), 0<*c*<1, the solution is (2.3). If λ_1_(*μ*)>λ_2_(*μ*), this solution tends to *Q*_1_(*μ*) as *t* goes to ∞ and to *Q*_2_(*μ*) when *t* goes to −∞. So, there is a heteroclinic connection from *Q*_2_(*μ*) to *Q*_1_(*μ*). In a similar way, if λ_1_(*μ*)<λ_2_(*μ*), a heteroclinic connection from *Q*_1_(*μ*) to *Q*_2_(*μ*) exists. ▪

**Remark 2.2**

Assume that for *μ*=*μ*_c_ the matrix *A* has the structure $\left(\begin{array}{cc} B & 0\\ C & D \end{array}\right)$, with all of the entries non-negative and the dominant eigenvalues of *B* and *D* coinciding and equal to λ_1_. Then, for λ_1_ there exists **u**_1_, eigenvector of *B*, and **u**_2_, eigenvector of *D*, with non-negative components. It is clear that ${\text{v}}_{1}=\left(\begin{array}{c} 0\\ {\text{u}}_{2} \end{array}\right)$ is an eigenvector of *A* with non-negative components. To get a second independent eigenvector of *A* with eigenvalue λ_1_, one should look for ${\text{v}}_{2}=\left(\begin{array}{c} {\text{u}}_{1}\\ {\text{w}}_{2} \end{array}\right)$, where **w**_2_ must satisfy *C***u**_1_+*D***w**_2_=λ_1_**w**_2_. In general, if such a vector **w**_2_ exists, it could have some negative components. This is why we ask explicitly for the existence of **v**_2_ with all components non-negative in condition (1).

**Remark 2.3**

Beyond the condition on the structure of *A*, we require the condition that the dominant eigenvalues of *B* and *D* coincide. Furthermore, the existence of **w**_2_ requires (λ_1_*I*−*D*)**w**_2_=*C***w**_1_. As λ_1_ is a simple eigenvalue of *D*, the kernel of (λ_1_*I*−*D*) has dimension one. Hence, the condition reduces to ask that *C***w**_1_ has no component in that kernel. Summarizing, the request reduces to two scalar conditions (beyond the positivity or zero values of the components of **w**_2_). Therefore, the condition for the trans-heteroclinic bifurcation has codimension two in the equations of the family (2.1), if we assume that the matrix *A* has the structure given in remark 2.2, as it happens for the system (2) in the present study.

**Remark 2.4**

A trans-heteroclinic bifurcation can also appear under more degenerate conditions. The matrix *A* could have a dominant eigenvalue with algebraic and geometric multiplicity equal to 3 or higher, or to have, for instance, a dominant eigenvalue with algebraic multiplicity 4 and geometric multiplicity 2, with two 2×2 Jordan blocs, such that there are two independent solutions of the form $t\exp(\lambda_{1}t)$. This would also lead to a line of fixed points.

For simplicity in the exposition let us denote as *L* the line of fixed points which appears when *μ*=*μ*_c_, and *L*(*μ*) the invariant line for *μ* near *μ*_c_.

### 2.3. Ecological interpretation of matrix *A* and extension to other biological systems

Let us consider the variable $x=(\begin{array}{c} y\\ z \end{array})$ which evolves according to

2.4 $$\frac{dy}{dt}=By-\phi y,$$

and

2.5 $$\frac{dz}{dt}=Cy+Dz-\phi z.$$

Note that the linear part of the equation for variables *y*(*t*) do not depend at all on any variable of block *z*, while variables *z*(*t*) depend on both block variables *y* and *z*. The dynamical system given by equations (2.4) and (2.5) can be considered as a model of two different ecological communities which define each block. Considering that *B*, *C* and *D* need to have non-negative entries one can expect different possibilities for trans-heteroclinic bifurcations to occur in ecological communities in a chemostat. Keeping the competition term indicated by *ϕ*, and naming the entries of block *C* as *c*_*ij*_, some general cases where this bifurcation could be found can be listed:

— Case *c*_*ij*_=0 ∀*i*,*j*. *Purely competitive communities*. For this system, the dynamics corresponds to two communities with Malthusian replication competing for resources. Hence, according to our results, the trans-heteroclinic bifurcation could underlie the competitive exclusion principle (‘Gause’s principle’) among communities in chemostats (see [50]).
— Case *c*_*ij*_>0 ∀*i*,*j*. *Competitive communities with asymmetric linear facilitation*. The case where all of the elements of matrix *C* are positive may correspond to a facilitation process (e.g. production of nutrients) carried out by community *y* on *z* (and not the other way around, i.e. commensalism). This facilitation can allow community *z* to better reproduce.

The previous two cases consider all of the elements *c*_*ij*_ to be zero or positive to illustrate different types of ecological interactions among the species of each block (or community). All other cases given by all possible combinations of these values of the elements *c*_*ij*_ might define different types of interactions: competition (*c*_*ij*_=0) and/or facilitation (*c*_*ij*_>0) between the species of two communities in chemostats’ ecosystems.

### 2.4. The effect of a perturbation

The next question to analyse will be what happens to the solutions of the initial quasi-species model when a sufficiently small, arbitrary and smooth perturbation is added to the system.

Let us consider the perturbed system

2.6 $$\dot{\text{x}}=A\text{x}-\Phi (\text{x})\text{x}+\varepsilon G(\text{x}),$$

where, as before, *A* depends on a parameter *μ* and *G* is assumed to be normalized (e.g. ∥*G*∥=1) and with bounded differential ∥*DG*∥<*M* for some fixed *M*>0. The bounds are assumed to hold in the full simplex $\sum_{i=1}^{n}x_{i}=1,x_{j}\geq 0,j=1,\ldots ,n$. Anyway, for a local study of the trans-heteroclinic bifurcation, it would be enough to assume that these bounds hold in a vicinity of *L*.

Note that if we want to keep the condition **u**^T^**x**=1, the vector field *G*(**x**) must be replaced by *G*(**x**)−**u**^T^*G*(**x**)**x** and to be normalized after this modification.

First we analyse the stability properties of the fixed points for the unperturbed vector field *F*(**x**)=*A***x**−*Φ*(**x**)**x**, under the hypothesis of proposition 2.1. We shall denote the eigenvalues of *A* by λ_*i*_, skipping the explicit dependence on *μ* for simplicity.

We recall that for *μ*≠*μ*_c_ there are two fixed points *Q*_1_, *Q*_2_ associated to the eigenvectors **v**_1_,**v**_2_.

We claim that the eigenvalues of the linearization of the vector field *F* at *Q*_1_, *DF*(*Q*_1_), are −λ_1_,λ_2_−λ_1_,λ_*j*_−λ_1_, *j*=3,…,*n*. The eigenvalues of *DF*(*Q*_2_) are −λ_2_,λ_1_−λ_2_,λ_*j*_−λ_2_ *j*=3,…,*n*. Then, if λ_1_>λ_2_ the point *Q*_1_ is attracting and *Q*_2_ has a one-dimensional unstable manifold and a stable one of dimension *n*−1. If λ_1_<λ_2_, they exchange their roles.

To prove the claim we perform a change of variables to put *A* in Jordan normal form. Let **z**=*P*^−1^**x** where *P* is an *n*×*n* matrix such that *P*^−1^*AP*=*J*

$$J=\left(\begin{array}{ccc} \lambda_{1} & 0 & 0\\ 0 & \lambda_{2} & 0\\ 0 & 0 & J_{3} \end{array}\right),$$

where *J*_3_ contains the remaining Jordan blocks, each one of them being an upper triangular matrix. The system for the new variable **z** becomes

$$\dot{\text{z}}=J\text{z}-\hat{\Phi }(\text{z})\text{z},$$

where $\hat{\Phi }(\text{z})=\Phi (P\text{z})={\text{u}}^{T}AP\text{z}={\text{u}}^{T}PJ\text{z}$. Using that the first two columns of *P* are the normalized eigenvectors **v**_1_,**v**_2_ we get that the first two components of **u**^T^*P* are equal to 1. Therefore

$$\hat{\Phi }(\text{z})=\lambda_{1}z_{1}+\lambda_{2}z_{2}+\sum_{j=3}^{n}\beta_{j}z_{j},$$

for some constants *β*_*j*_, *j*=3,…,*n*. The differential of the vector field in **z** is $M(\text{z}):=J-\hat{\Phi }(\text{z})I_{n}-\text{z}D\hat{\Phi }(\text{z})$ where *I*_*n*_ denotes the identity in dimension *n*.

In the new variable **z** the coordinates of *Q*_1_ are *z*_1_=1, *z*_*j*_=0, *j*=2,…,*n*. It is easy to check that at *Q*_1_ the matrix M becomes an upper triangular matrix

$$\left(\begin{array}{ccc} -\lambda_{1} & \lambda_{2} & \ldots \\ 0 & \lambda_{2}-\lambda_{1} & \ldots \\ 0 & 0 & J_{3}-\lambda_{1}{\mathrm{Id}}_{n-2} \end{array}\right),$$

and then the eigenvalues are −λ_1_,λ_2_−λ_1_,λ_*j*_−λ_1_, *j*=3,…,*n*.

The eigenvalues at *Q*_2_ are computed in the same way taking into account that *Q*_2_ has coordinates *z*_1_=0,*z*_2_=1,*z*_*j*_=0, *j*=3…,*n*.

When we return to the value *μ*=*μ*_c_ with λ_2_=λ_1_, instead of a single fixed point one has the line of fixed points *L*. A fixed point *Q* in that line has *z* coordinates as *z*_1_=*α*, *z*_2_=1−*α*, *z*_*j*_=0, *j*=3,…,*n*, for some real *α*. Then M becomes

$$\left(\begin{array}{ccc} -\alpha \lambda_{1} & -\lambda_{2} & \ldots \\ -(1-\alpha )\lambda_{1} & -(1-\alpha )\lambda_{1} & \ldots \\ 0 & 0 & J_{3}-\lambda_{1}{\mathrm{Id}}_{n-2} \end{array}\right).$$

Therefore, the eigenvalues of the differential are 0,−λ_1_,λ_*j*_−λ_1_,*j*=3,…,*n*. Beyond the 0 due to the existence of the line of fixed points, *L*, all the other eigenvalues have negative real part if the assumptions of proposition 2.1 hold.

The previously stated facts say that *L* is a normally hyperbolic invariant manifold (hereafter NHIM). That is, at the points of *L* the space $R^{n}$ splits as a one-dimensional space associated to the eigenvalue 0 (i.e. in the direction of *L*) and an (*n*−1)-dimensional space associated to all the eigenvalues with negative real part.

It is well known (see, e.g. [51] or [52]) that NHIM persist under sufficiently small perturbation. This shows that for the system *F*+*εG* there exists an invariant curve ${\hat{L}}_{\varepsilon }$, close to *L* and also normally hyperbolic. And the same happens if one takes into account variations of *μ* close to *μ*_c_. We summarize these results as follows:

**Proposition 2.5**

*Let us consider the system* (*2.6*) *with* *A*(*μ*) *satisfying the hypothesis of proposition* 2.1*, the normalization of* *G* *and a bound on* ∥*DG*∥*, as mentioned. Then, if* *ε* *is sufficiently small, for* *μ* *in a vicinity of* *μ*_c_*, there exists an attracting invariant curve* ${\hat{L}}_{\varepsilon }(\mu )$*, close to* *L*(*μ*).

### 2.5. Robustness of the trans-heteroclinic bifurcation under perturbations

The final question to consider is what happens to the dynamics in the new invariant attracting curve ${\hat{L}}_{\varepsilon }(\mu )$ for *μ* near *μ*_c_. That is, we want to see the robustness of the trans-heteroclinic bifurcation when a perturbation is added to the quasi-species model.

**Proposition 2.6**

*Let us consider the system* (*2.6*) *with* *A*(*μ*) *satisfying the hypothesis of proposition* 2.1. *Let* *μ*_1_=*μ*_c_−Δ *and* *μ*_2_=*μ*_c_+Δ *for some* Δ>0 *small enough. Assume that* *KΔ*<|λ_1_(*μ*_*j*_)−λ_2_(*μ*_*j*_)|, *j*=1,2, *for some given positive* *K*.

*Then for* *ε* *sufficiently small, in* ${\hat{L}}_{\varepsilon }(\mu )$ *there exists a heteroclinic connection from* *Q*_1_(*ε*) *to* *Q*_2_(*ε*) *if* *μ*=*μ*_1_ *and, a heteroclinic connection from* *Q*_2_(*ε*) *to* *Q*_1_(*ε*) *if* *μ*=*μ*_2_.

**Remark 2.7**

For a small fixed value of *ε* and Δ small enough, that is for *μ* close enough to *μ*_c_, the dynamics on the invariant curve ${\hat{L}}_{\varepsilon }(\mu )$ can have other fixed points or, eventually, no fixed points at all.

**Proof.**

For simplicity we skip the explicit dependence on *μ*. If *ε*=0 and *μ*=*μ*_2_, the fixed points *Q*_1_ and *Q*_2_ are the only ones which remain from the line of fixed points *L*. As has been mentioned, the eigenvalues of *DF*(*Q*_1_) are λ_*i*_−λ_1_ for *i*=2,3,…,*n* and −λ_1_, showing that *Q*_1_ is an attractor. The eigenvalues at *DF*(*Q*_2_) are λ_*i*_−λ_2_ for *i*=1,3,…,*n* and −λ_2_, showing that *Q*_2_ has a one-dimensional unstable manifold. It gives the connection. The same happens for *μ*=*μ*_1_, exchanging the role of the points.

Now let us consider the case *ε*≠0. By assumption all the eigenvalues of *DF*(*Q*_1_) and *DF*(*Q*_2_) are bounded away from zero by *KΔ*, for Δ>0 small enough. Here we use also the fact that the eigenvalues λ_1_ and λ_2_ are at a finite distance of λ_*j*_, *J*≥3, according to assumption (2) in proposition 2.1. Hence, the differential matrices are regular, and the implicit function theorem applied to *F*+*εG* ensures that there exist single fixed points *Q*_1_(*ε*) and *Q*_2_(*ε*) for *F*+*εG* if *ε* is sufficiently small, in some neighbourhoods around *Q*_1_ and *Q*_2_. In the part of ${\hat{L}}_{\varepsilon }(\mu )$ between *Q*_1_(*ε*) and *Q*_2_(*ε*) which excludes these neighbourhoods the field *F* is bounded away from zero and, hence, no new fixed points can appear for *F*+*εG* on it when *ε*≠0 is sufficiently small because of the bound on ∥*G*∥. Furthermore, because of the bound on ∥*DG*∥ the dominant eigenvalues of the differential at *Q*_1_(*ε*) and *Q*_2_(*ε*) change only slightly and the dimensions of the manifolds at these points, are preserved if *ε* is sufficiently small. ▪

Finally, let us consider the case Δ close to zero (including the case *μ*=*μ*_c_, i.e. Δ=0). By the properties of NHIM under perturbation, the line *L* will be replaced by a nearby (at distance O(ε)) invariant curve ${\hat{L}}_{\varepsilon }$. The dynamics on ${\hat{L}}_{\varepsilon }$ depends on the perturbation *G* and can be of any of the types which appear in one-dimensional flows: all the points move (no fixed points), either in one direction or the other; several fixed points with alternative stability properties show up; some points can be at a saddle–node bifurcation, and the like.

As a conclusion, except for a narrow interval of *μ* around *μ*_c_ whose size is of the order of *ε*, the change from heteroclinic connection from *Q*_2_(*ε*) to *Q*_1_(*ε*) to a heteroclinic connection from *Q*_1_(*ε*) to *Q*_2_(*ε*), persists. As an example we look at the model considered here given by equations (2.2) and perturb with a vector field *εG* such that all the components of *G* are zero except the first one, which is taken as $g(x_{1})=\cos(15x_{1})$. As done in some examples in [44] we take the parameter values *r*=0.1 and *δ*_*r*_=0.05, which give $\mu_{c}=\frac{1}{3}$ and *δ*_*μ*_=0.05. For coherence with the present notation we rename the variables *x*_0_,…,*x*_7_ used in [44] as *x*_1_,…,*x*_8_, we also assume that the condition **u**^T^**x**=1 is satisfied for all time if it is satisfied at *t*=0 and we use the notation $P_{2}^{*}(\varepsilon ),P_{3}^{*}(\varepsilon )$ for the fixed points. We recall that for *ε*=0 the first components of $P_{2}^{*}$ and $P_{3}^{*}$ are, respectively, *x*_1_=1 and *x*_1_=0. The perturbation *G* can be modified, as explained before, to satisfy **u**^T^**x**=1 for all time or, alternatively, we redefine the function *Φ* in the equations as

$$\Phi =r(x_{1}+x_{3})+\hat{r}(x_{5}+x_{7})+\varepsilon g(x_{1}),$$

where $\hat{r}=r+\delta_{r}$. In particular this implies that $P_{2}^{*}$ is not changing when *μ* and *ε* are changed.

**Figure 3.**
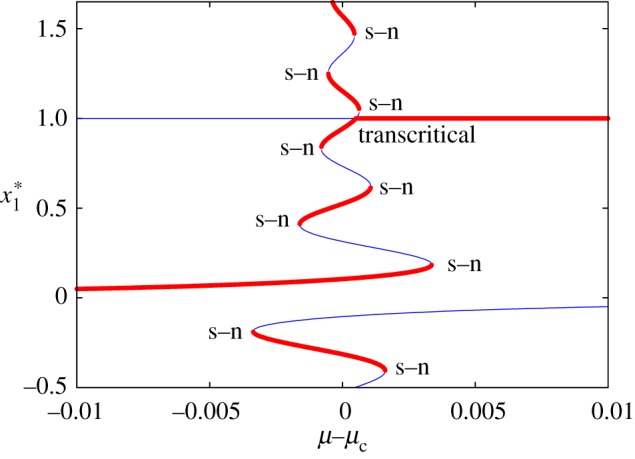
Fixed points and bifurcations identified for the perturbed model. In the horizontal axis we plot $\mu -\frac{1}{3}$ and in the vertical axis the *x*_1_ coordinate of the fixed points $\left(x_{1}^{*}\right)$. Blue (red) color corresponds to unstable (stable) fixed points. Hence, the dynamics on the invariant line, projected on the *x*_1_ variable and for every fixed value of *μ*, is going away from the points in blue and tending to the points in red. Several bifurcations such as saddle–node (s–n) or transcritical are indicated.

Figure 3 shows the fixed points in this case as a function of *μ*, setting *ε*=10^−4^. Specifically, this figure shows that for *μ*−*μ*_c_<−0.003366 there are only two fixed points $P_{2}^{*}$ and $P_{3}^{*}(\varepsilon )$ in ${\hat{L}}_{\varepsilon }$ and, so, there is only a heteroclinic connection from $P_{2}^{*}$ to $P_{3}^{*}(\varepsilon )$. This connection subsists until *μ*−*μ*_c_≈−0.00161, together with other new fixed points. One can also see several saddle–node (s–n) bifurcations and a transcritical bifurcation at *x*_1_=1. Several solutions have no meaning in the context of the biological model because some of the variables can be negative or greater than 1. It is easy to check (both theoretically and numerically) that the size of the domain in *μ* for which the heteroclinic connection between $P_{2}^{*}$ and $P_{3}^{*}(\varepsilon )$ do not exists is O(ε).

**Figure 4.**
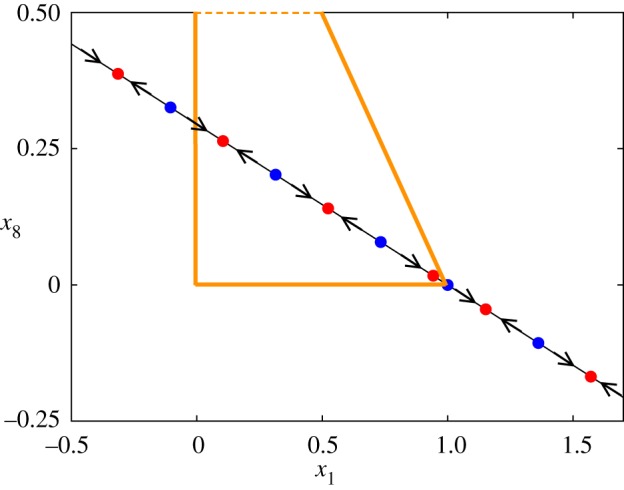
Invariant line at bifurcation value for the perturbed model. The invariant curve for $\mu =\frac{1}{3}=\mu_{c}$ seen in the (*x*_1_,*x*_8_) variables. Blue (red) points correspond to unstable (stable) equilibria. The area framed with the orange lines is the biologically-meaningful phase space given by the simplex.

Figure 4 displays for *μ*=*μ*_c_ the fixed points on the line ${\hat{L}}_{\varepsilon }$ which in the present example and for *μ*=*μ*_c_ coincides with *L*. The plot shows the variables (*x*_1_,*x*_8_). As before the blue (red) points correspond to unstable (stable) fixed points. Only six of them play a role if we are interested in *x*_1_∈[0,1]. From every unstable point there are heteroclinic connections to the two nearby stable points.

## 3. Discussion

In a recent article [44], we identified a novel bifurcation involving a catastrophic shift. This bifurcation, which is global and was named as a *trans-heteroclinic bifurcation*, was found in the quasi-species equation [45], used to investigate the dynamics of competition between healthy and tumour heterogeneous cells given by different phenotypes [44]. Starting from this context, and to provide a complete description of this new bifurcation, we have here developed a thorough analysis of the mechanisms responsible for this bifurcation, paying special attention to the structure of the replication–mutation matrix of Eigen’s quasi-species model where this type of bifurcation is found.

The trans-heteroclinic bifurcation causes a catastrophic shift since the exchange of stability between two fixed points is produced without their collision, in contrast with the transcritical and the saddle–node bifurcations [23]. Hence, systems with trans-heteroclinic transitions are not bistable because each one of the two equilibria, when stable, is globally asymptotically stable. We have also analysed the effect of small perturbations at the bifurcation value, where a line of fixed points lives within the simplex.

For a general ordinary differential equation, the fact that it has a line of fixed points is a codimension infinity bifurcation, i.e. is extremely degenerate. However, in the present case, inside the quasi-species models and assuming that the matrix *A* is irreducible, is just a codimension two bifurcation (see remark 2.4).

We have noticed that this type of transition is also found in a quasi-species mathematical model describing the so-called survival-of-the-flattest effect (see [49]). The survival-of-the-flattest effect, described experimentally in viroids [53] and in competing computer programs [54], involves the survival of slow-reproducing replicons that are robust against deleterious mutations, and the vanishing of fast-reproducing ones that are not robust to deleterious mutations. Specifically, the model studied in [49] presented a discontinuous transition when increasing the mutation rate that involved the outcompetition of the fast-replicating, fit quasi-species by the slow-replicating, flat one. The replication–mutation matrix for this model fulfils the conditions for the presence of the trans-heteroclinic bifurcation discussed in this article (see electronic supplementary material, §2).

It is known that transients typically suffer long delays at the vicinity of bifurcations. For instance, near a saddle–node bifurcation occurring at a critical value *μ*_c_ and assuming that these fixed points exist for *μ*<*μ*_c_, the delay, named delayed transition, is $\tau =O({\left(\mu_{c}-\mu \right)}^{-1/2})$. This scaling law, identified in different nonlinear systems such as charge density waves and hypercycles [23,25,55], has been experimentally characterized in electronic circuits [2]. For transcritical bifurcations one has $\tau =O(|\mu -\mu_{c}{|}^{-1})$, and the delay is similar for a pitchfork bifurcation, when an attracting point, until *μ*=*μ*_c_, becomes unstable for *μ*>*μ*_c_ and two nearby stable points appear. In this case $\tau =O({\left(\mu -\mu_{c}\right)}^{-1})$. Pitchfork bifurcations have been recently identified in experiments with elastic structures [4]. The trans-heteroclinic bifurcation has the same type of behaviour: $\tau =O(|\mu -\mu_{c}{|}^{-1})$, both for *μ*<*μ*_c_ and for *μ*>*μ*_c_. The derivation follows immediately from [44] (see also electronic supplementary material, §3).

Summarizing, the bifurcation described in this article, identified in a family of quasi-species equations [45], involves a new type of catastrophic (abrupt) transition arising within the framework of evolutionary biology. The quasi-species model provides a very powerful mathematical framework to investigate the evolutionary dynamics of biological information. Initially conceived to explore the dynamics of prebiotic replicons [45], quasi-species theory has been widely used to investigate the dynamics of RNA viruses [56–60], cancer evolution [44,61,62] and compositional assemblies [63]. The results of this article provide the conditions for which catastrophic shifts due to trans-heteroclinic bifurcations should be expected in quasi-species systems, also introducing a novel type of bifurcations in nonlinear dynamical systems. Indeed, our results can be extended to any system with (exponentially) self-replicating species in a chemostat due to the generality of equation (2.1).

## Supplementary Material

Supporting Information

## Data accessibility

All data are including in the article and in the electronic supplementary material.
