# Supplementary material for: Trans-heteroclinic bifurcation: a novel type of catastrophic shift
Source: R Soc Open Sci. 2018 Jan 24;5(1):171304. doi: 10.1098/rsos.171304 (PMC5792914; doi:10.1098/rsos.171304)
Supplement: Supporting Information [file rsos171304supp1.pdf]

Trans-heteroclinic bifurcation: a novel type of catastrophic shift Supporting Information

Josep Sardanyés[1,2] Regina Martínez[3] Carles Simó[4,2],
1.Centre de Recerca Matemàtica, Edifici C, 08193 Bellaterra, Barcelona, Spain
2. Barcelona Graduate School of Mathematics (BGSMath) Edifici C, 08193 Bellaterra, Barcelona, Spain
3. Departament de Matemàtiques, Edifici C, Universitat Autònoma de Barcelona 08193 Bellaterra, Spain
4. Departament de Matemàtiques i Informàtica (Universitat de Barcelona),
Gran Via de les Corts Catalanes 585, 08007 Barcelona, Spain

1. QUASISPECIES MODELS WITH CONSTANT POPULATION: DYNAMICS IN CHEMOSTATS

Eigen's original model on quasispecies used the so-called constant population (CP) constraint **???**. This approach assumes that self-replicating molecules live in a well-stirred tank reactor (chemostat) and their population is kept constant by means of a dilution outflux. The CP constraint bounds the growth of the macromolecules also introducing competition between all of the components of the tank, which compete for available resources (e.g., building blocks or mononucleotides). Within the framework of origins of life and prebiotic evolution, Eigen conceived the quasispecies model to investigate the dynamics of biological information. The quasispecies model provides a formal description of the processes of Darwinian evolution of self-replicating entities. A quasispecies is a cloud of related genotypes that exist under high mutation rates, where a large fraction of offspring are expected to contain one or several mutations relative to the parent **?**. The quasispecies model usually considers two coupled processes, replication and mutation, following the next two reactions:

- error-free replication of template I_i (that is, a duplication):

$$(s) + I_i \xrightarrow{f_i Q_{ii}} 2I_i \ ,$$

- erroneous replication of template I_i giving place to template $I_{j\neq i}$:

$$(s) + I_i \xrightarrow{f_i Q_{ji}} I_i + I_j \ .$$

The symbol s denotes building blocks (e.g., mononucleotides) that are required for replication and that are not considered explicitly. Error-free replication and mutation are parallel reactions of the same mechanism. The rate of replication, f_i, depends on template I_i; the mutation probability Q_{ji} depends on both the template and the product of the replication. We note that $\sum_{j=1}^{n} Q_{ji} = 1, \forall i$. In addition, we consider an unspecific degradation or dilution flow $I_i \to 0$, which may be adjusted in such a way that the total population is of constant size. Here we describe different quasispecies models with different replication-mutation matrices, A, according to the general quasispecies model. The matrix A here is given by $a_i Q_{ji}$, thus containing both fitness and mutation terms of sequence i. The general model is given by:

$$\frac{d\mathbf{x}}{dt} = A\mathbf{x} - \Phi(\mathbf{x})\mathbf{x}, \qquad (1)$$

being $\mathbf{x} = (x_0, x_1, ..., x_n)$ the vector of population densities for n different sequences. Here $\Phi(\mathbf{x})$ denotes the outflow term, which keeps the population constant. The CP assumption implies that $\sum_i^n x_i = C$ (usually with $C = 1$) and since the population is maintained constant $\sum_i^n \dot{x}_i = 0$.

Matrix A corresponds to the so-called replication-mutation matrix, and contains the replication rates (f_i) and mutation probabilities (Q_{ji}). This matrix can be represented, in its general form, by:

$$A = \begin{pmatrix} f_1 Q_{11} & f_2 Q_{12} & \cdots & f_n Q_{1n} \\ f_1 Q_{21} & f_2 Q_{22} & \cdots & f_n Q_{2n} \\ \vdots & \vdots & \ddots & \vdots \\ f_1 Q_{n1} & f_2 Q_{n2} & \cdots & f_n Q_{nn} \end{pmatrix}.$$

In particular if some $f_i = 0$ the related column consists of zeros. Notice that the mutation terms in the diagonal of matrix A indicate the error-free replication, since they are of the form Q_{ii} (i.e., a sequence i produces another sequence i, an exact copy). All of the other terms denote the mutation processes giving sequence i from sequence j by means of Q_{ij}. Depending on the fitness landscape, the matrix A will have different structures. We notice that for models neglecting backward mutations, the

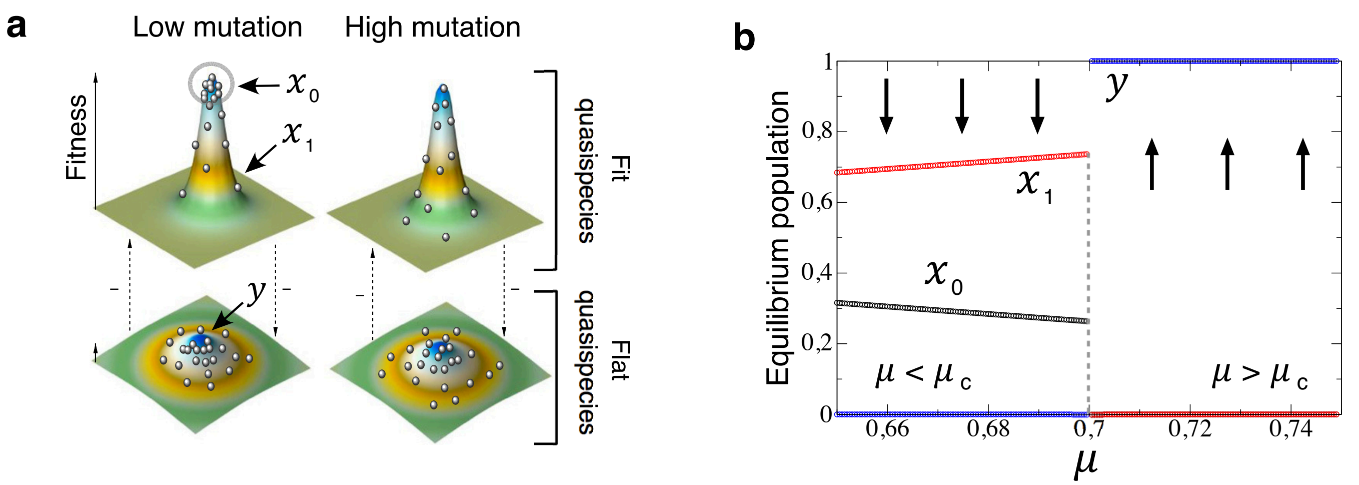

FIG. 1. **Trans-heteroclinic bifurcation in a model for the survival-of-the-flattest**. Schematic diagram of the survival-of-the-flattest effect, where two different quasispecies compete. The fit quasispecies is located on a sharp, single-peak fitness landscape, while the second quasispecies is located on a flat, single-peak landscape (see **?**). (b) Bifurcation diagram for Eqs. (5) obtained increasing mutation rate, μ, using $f_x^{(0)} = 1$, $f_x^{(1)} = 0.05$, $f_y = 0.3$, and $\epsilon = 0$. Here, once a critical mutation rate (μ_c = 0.7) is overcome the system suffers a trans-heteroclinic bifurcation. We note that the bifurcation value as well as the values of the fixed points do not depend on ϵ.

replication-mutation matrix is triangular. Equation (1) can indeed be applied to investigate the dynamics of any system of self-replicating entities (Malthusian replicators) within a chemostat **??**. Chemostats have been used to characterize experimentally the long-term evolutionary dynamics of replicating species such as bacteria **?** or yeast **???**. In the case of Eigen model **?**, equation (1) can be expressed as:

$$\frac{dx_i}{dt} = \sum_j Q_{ij} f_j x_j - x_i \left(\sum_j f_j x_j \right), \qquad (2)$$

where the matrix element Q_{ij} describes the transition probabilities from the type j to the type i. The elements of mutation matrix are $Q_{ij} = q^{L-d(j,i)}(1-q)^{d(j,i)}$. Here q is the probability of error-free replication per nucleotide and replication cycle; L is the length of the sequence (in number of nucleotides or bits); and $d(j,i)$ is the Hamming distance between sequences j and i; the diagonal terms of the mutation matrix are $Q_{ii} = q^L \equiv Q \equiv e^{-\gamma}$, where $\gamma = -L\ln(q) \approx N(1-q)$ is the parameter of mutation in the Eigen model and f_i is identified as a fitness (replication rate) of the sequence i. Here, x_i satisfy normalization condition $\sum_i x_i = 1$. Within the fields of viral **??** and cancer **??** quasispecies, and when the length of the sequences is not explicitly introduced, Eqs. (1) and (2) can be also expressed as:

$$\dot{x}_i = (1-\mu) f_i x_i + \mu \sum_{<j>_i} f_j x_j - x_i \Phi(\mathbf{x}). \qquad (3)$$

Here f_i is again the replication rate (fitness) of sequence i and μ is the mutation rate (often also expressed as $Q = 1 - \mu$, Q being the quality replication factor). Notice that here mutation rates are homogeneous for all sequences and differences only arise in the replication rates, which determine the shape of the fitness landscape. The first RHS term of Eq. (3) is the error-free replication of sequence i, while the second RHS term is the production of sequence i due to mutation of the nearest neighboring sequences j (represented by $< j >_i$) of the sequence space.

2. THE TRANS-HETEROCLINIC BIFURCATION IN THE SURVIVAL-OF-THE-FLATTEST

1. Differential equations model

In this section we introduce a mathematical model describing the dynamics of the survival-of-the-flattest effect, analyzed in ref. **?**, which also presents a trans-heteroclinic bifurcation. This model describes the dynamics between a fit quasispecies (x) located at a sharp, single-peak fitness landscape competing with a less fit quasispecies (y) that is placed at a flatter single-peak fitness landscape (see Fig. 1a). Under this scenario, quasispecies x replicates faster but it is less robust to mutation. On the

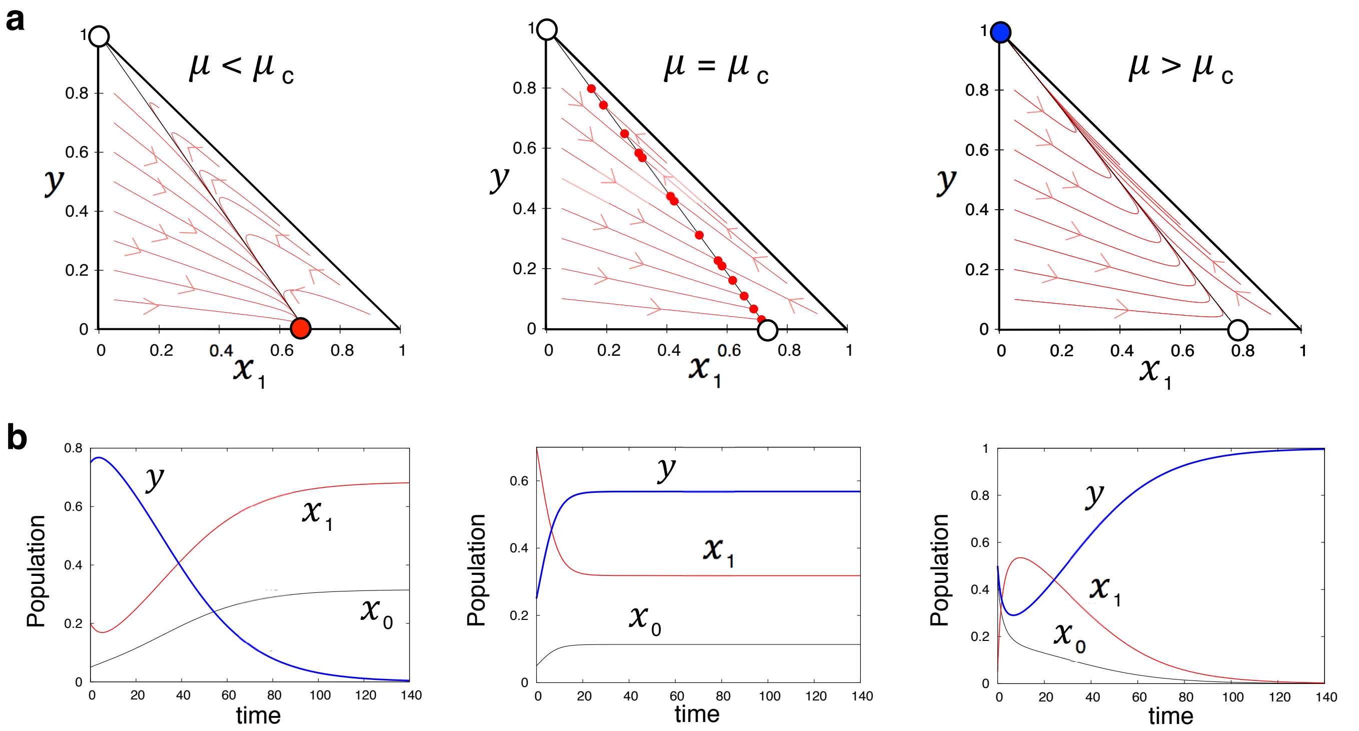

FIG. 2. **Topological changes and dynamics tied to the trans-heteroclinic bifurcation in the survival-of-the-flattest.** (a) Phase portraits with $\mu = 0.65 < \mu_c$ (left); $\mu = \mu_c = 0.7$ (middle); and $\mu_c < \mu = 0.75$ (right) using the same parameter values as in (b). The arrows indicate the direction of the orbits. Stable fixed points are indicated with solid circles while unstable ones are indicated with white circles. The black line inside the simplexes corresponds to the heteroclinic connection. (b) Time series for each case represented in the phase portraits obtained, in each panel, with a single initial condition. In all of the panels we used $f_x^{(0)} = 1$, $f_x^{(1)} = 0.05$, $f_y = 0.3$, and $\epsilon = 0$.

contrary, the flat quasispecies displays a lower fitness is more robust to deleterious mutations. With the aim of introducing a minimal model to analyze the survival-of-the-flattest effect, a master sequence and the pool of mutants were used as state variables. Under the constant population constraint assumption $\sum_i (x_i + y_i) = 1$, the model was defined by the following set of ODEs:

$$\begin{aligned}
\frac{dx_0}{dt} &= f_x^{(0)} Q x_0 - x_0(\mathbf{\Phi} + \epsilon), \\
\frac{dx_1}{dt} &= f_x^{(0)}(1-Q)x_0 + f_x^{(1)} x_1 - x_1(\mathbf{\Phi} + \epsilon), \\
\frac{dy_0}{dt} &= f_y^{(0)} Q y_0 + f_y^{(1)} y_1 (1-Q) - y_0(\mathbf{\Phi} + \epsilon), \\
\frac{dy_1}{dt} &= f_y^{(1)} Q y_1 + f_y^{(0)} y_0 (1-Q) - y_1(\mathbf{\Phi} + \epsilon).
\end{aligned} \tag{4}$$

The outflow term is $\mathbf{\Phi} = \sum_{i=0,1} [(f_x^{(i)} - \epsilon)x_i + (f_y^{(i)} - \epsilon)y_i]$. Constants $f_x^{(0)} > 0$ and $f_y^{(0)} > 0$ are the replication rates of the master fit and master flat sequences, respectively. Parameters $f_x^{(1)} \geq 0$ and $f_y^{(1)} > 0$ are, respectively, the replication rates of both mutant sequences for the fit and the flat quasispecies. Here constant Q denotes the average copying fidelity, being $\mu = 1 - Q$ the mutation rate. Finally, $0 \leq \epsilon \ll 1$ is a degradation rate, assumed to be equal for all of the sequences. The replication-mutation matrix A for the system Eqs. (4) reads:

$$A = \begin{pmatrix} f_x^{(0)} Q - \epsilon & 0 & 0 & 0 \\ f_x^{(0)}(1-Q) & f_x^{(1)} - \epsilon & 0 & 0 \\ 0 & 0 & f_y^{(0)} Q - \epsilon & f_y^{(1)}(1-Q) \\ 0 & 0 & f_y^{(0)}(1-Q) & f_y^{(1)} Q - \epsilon \end{pmatrix},$$

To study the survival-of-the-flattest effect we need to set the following parameter relations: $f_x^{(0)} \gg f_x^{(1)}$; and $f_x^{(1)} < f_y^{(1)} < f_y^{(0)} < f_x^{(0)}$. The previous conditions reproduce the shape of the fitness landscapes displayed in Fig. 1a, considering that the

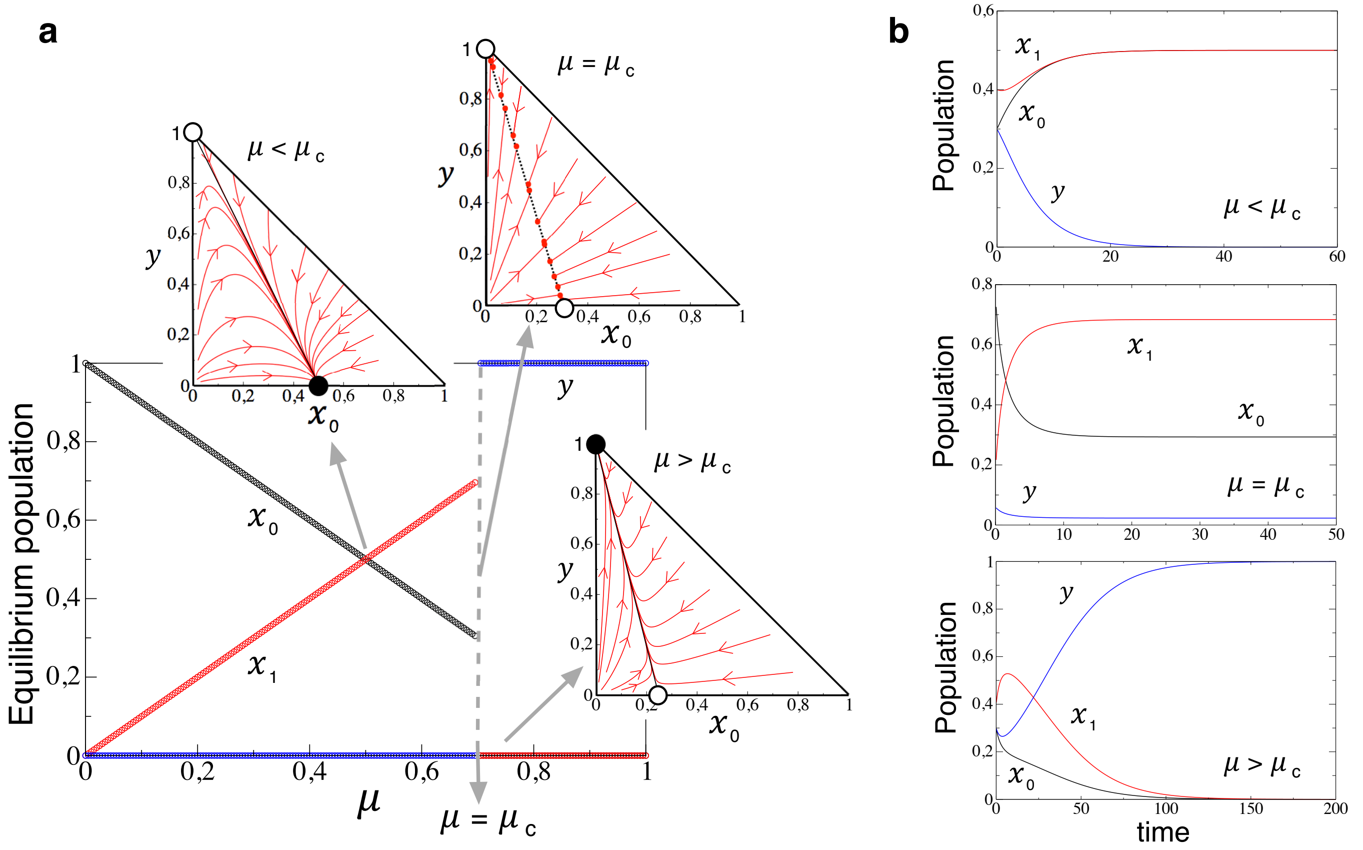

FIG. 3. **The trans-heteroclinic bifurcation in the survival-of-the-flattest with lethal mutants.** (a) Bifurcation diagram considering lethal mutants for the fit quasispecies, setting $f_x^{(1)} = 0$. A trans-heteroclinic bifurcation is found at $\mu = \mu_c = 0.7$. The simplex display (from left to right) the orbits with $\mu = 0.5 < \mu_c$, $\mu = \mu_c = 0.7$, and $\mu_c < \mu = 0.75$. Stable and unstable fixed points for values below and above the bifurcation are indicated with black and white circles, respectively. (b) Time series for each case represented with the phase portraits in (a) using a single initial condition. Here we also set $f_x^{(0)} = 1$, $f_y = 0.3$, and $\epsilon = 0$.

mutant and the fit replicators of the flat quasispecies have differential fitnesses. However, the dynamical system given by Eqs. (4) can be reduced to a three-variable model by considering that the changes in fitness due to mutations for the flat quasispecies are very small. Under this assumption, both master and mutant variables for this quasispecies can be lumped together **?**. Hence, by considering that $y = y_0 + y_1$, and $f_y = f_y^{(0)} = f_y^{(1)}$ (now with $f_x^{(1)} < f_y < f_x^{(0)}$), and thus $\dot{y} = \dot{y}_0 + \dot{y}_1$, the model can be simplified to:

$$\begin{aligned}
\frac{dx_0}{dt} &= f_x^{(0)} Q x_0 - x_0 \mathbf{\Phi} - \epsilon x_0, \\
\frac{dx_1}{dt} &= f_x^{(0)} (1-Q) x_0 + f_x^{(1)} x_1 - x_1 \mathbf{\Phi} - \epsilon x_1, \\
\frac{dy}{dt} &= f_y y - y(\mathbf{\Phi} + \epsilon).
\end{aligned} \tag{5}$$

Matrix A for this case reads:

$$A = \begin{pmatrix} f_x^{(0)} Q - \epsilon & 0 & 0 \\ f_x^{(0)}(1-Q) & f_x^{(1)} - \epsilon & 0 \\ 0 & 0 & f_y - \epsilon \end{pmatrix},$$

The previous model considered deleterious mutants for the fit quasispecies, since $f_x^{(1)} < f_x^{(0)}$. Another interesting case that also presents the trans-heteroclinic bifurcation is to consider that mutants are lethal i.e., $f_x^{(1)} = 0$. For this slightly different

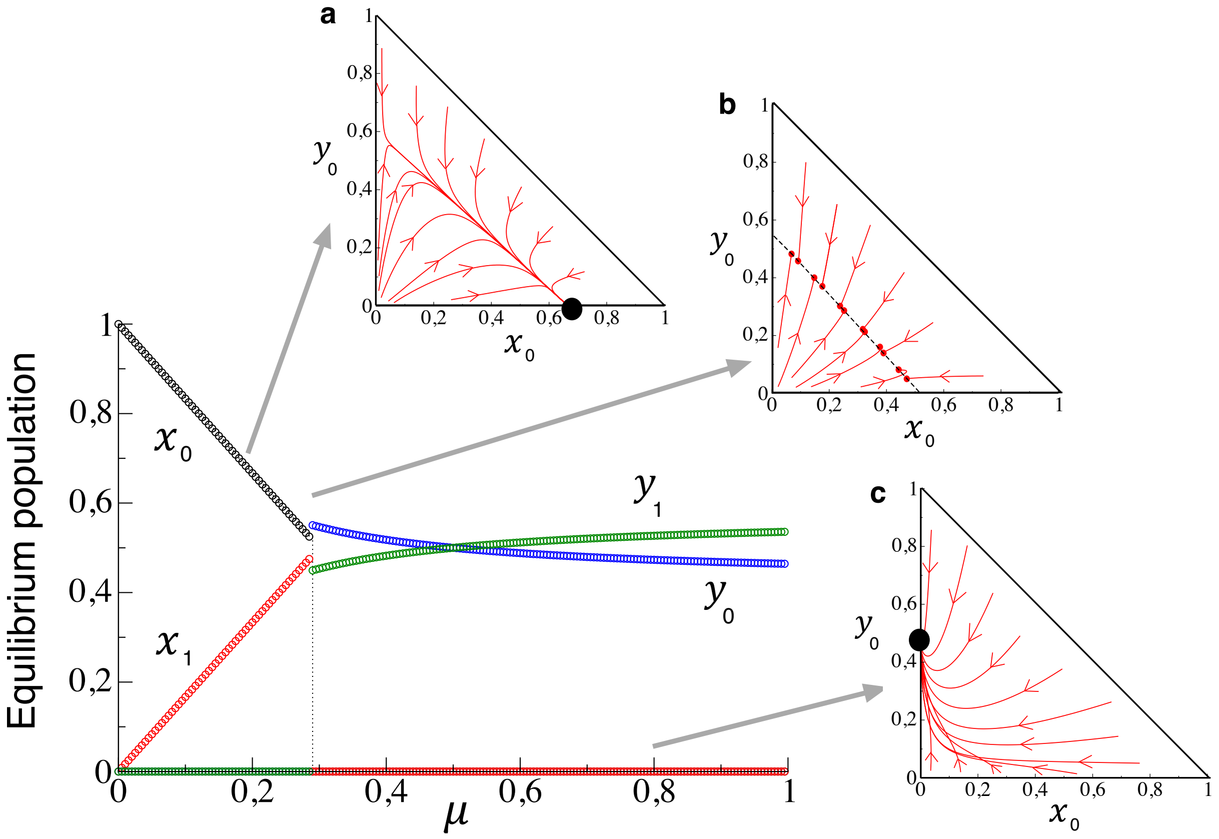

FIG. 4. **Bifurcation diagram and phase portraits for the four-variable quasispecies model given by Eqs. (4).** (a) Catastrophic shift at increasing μ, considering $f_x^{(0)} = 1, f_x^{(0)} = 0.4, f_y(0) = 0.8, f_y^{(1)} = 0.6$ and $\epsilon = 0.1$. The trans-heteroclinic bifurcation is found at $\mu = \mu_c = 0.28989795\cdots$. The phase portraits display the orbits projected in the simplex (x_0, y_0) at (a) $\mu = 0.2 < \mu_c$; (b) $\mu = \mu_c$; and (d) $\mu_c < \mu = 0.8$. Stable fixed points are indicated with black circles. Here as well the equilibrium values do not depend on ϵ.

fitness landscape, matrix A reads:

$$A = \begin{pmatrix} f_x^{(0)}Q - \epsilon & 0 & 0 \\ f_x^{(0)}(1-Q) & -\epsilon & 0 \\ 0 & 0 & f_y - \epsilon \end{pmatrix},$$

2. Dynamics and critical thresholds governed by the trans-heteroclinic bifurcation

Let us first explore the simpler case given by equations (5). This system has four fixed points, given by $P_0^* = (0,0,0)$, $P_1^* = (0,0,1)$, $P_2^* = (0,1,0)$, and $P_3^* = (x_0^*, 1 - x_0^*, 0)$, with

$$x_0^* = \frac{f_x^{(0)}Q - f_x^{(1)}}{f_x^{(0)} - f_x^{(1)}}.$$

Notice that the fixed point P_0^* is not meaningful under the CP assumption since the sum of all of the variables must be equal to 1. The other fixed points have the following biological meanings: when P_1^* is stable, the flat quasispecies outcomptes the fit quasispecies, while when $P_{2,3}^*$ are stable, the fit quasispecies outcompetes the flat one. P_2^* involves that the fit quasispecies is completely composed of mutant sequences, while P_3^* ensures coexistence between the master and the mutant sequences of the fit quasispecies. A sharp bifurcation when mutation rate is increased was characterized for system Eqs. (5) (see **?**). For this

reduced model the bifurcation value is given by

$$\mu_c = 1 - \frac{f_y}{f_x^{(0)}}. \tag{6}$$

The bifurcation diagram for Eqs. (5) obtained increasing mutation rate, μ, is displayed in Fig. 1b. Below the critical mutation rate, $\mu_c = 0.7$, the fit quasispecies ($x_{0,1}$) outcompete the flat one at equilibrium. However, a sharp transition occurs at $\mu = \mu_c = 0.7$, given by a trans-heteroclinic bifurcation. Above this bifurcation, the flat quasispecies is the one dominating the entire population. The topological changes of the phase space for the bifurcation scenario displayed in Fig. 1b are illustrated in Fig. 2. Here, we represent projections in the simplex (x_1, y), and the fixed points involved in the bifurcation are $P_1^* = (0, 0, 1)$ and $P_3^* = (x_0^*, 1 - x_0^*, 0)$. When $\mu < \mu_c$, P_1^* is unstable, and the orbits rapidly evolve towards P_3^*. Notice that the orbits reach the heteroclinic connection indicated with a thin black line joining the two fixed points. At the bifurcation value, as happened for the model analyzed in the main manuscript (see Fig. 2 in the main manuscript and **?**), the equilibrium state inside the simplex is given by a line of fixed points that substitute the heteroclinic connection found when $\mu \neq \mu_c$. The time dynamics at the bifurcation value involves a rapid approach to this line of fixed points, and the equilibrium population depends on the initial conditions, as a difference from values of $\mu \neq \mu_c$, where the orbits reach an asymptotically, globally stable fixed point (either P_1^* or P_3^* depending on μ, see the time series in Fig. 2). After the bifurcation, the heteroclinic connection is recovered but the stability nature of fixed points P_1^* and P_3^* is interchanged.

Figure 3a displays the bifurcation diagram and the dynamics considering lethal mutants (also with $\epsilon = 0$). Here we are also interested in the fixed points $P_1^* = (0, 0, 1)$ and $P_3^* = (x_0^*, 1 - x_0^*, 0)$, where $x_0^* = Q$ for $f_x^{(1)} = 0$. The trans-heteroclinic bifurcation for this case also takes place when $\mu = \mu_c = 0.7$. Three different simplexes are displayed below, at, and above the bifurcation. Notice that here we used the projections (x_0, y). Since the mutant fit species do not replicate, there exists a smaller interference on the growth of the fit master sequence. Then, the coordinate x_0^* of the fixed point P_3^* is larger. Some time series are also displayed in Fig. 3b. Notice that when $\mu = 0.5 < \mu_c$, the populations of x_0 and x_1 achieve the same equilibrium value. At the bifurcation value, as previously discussed, the population equilibrium depends on the initial conditions.

Now we provide some insights into the full model considering the four variables. It is easy to show that the critical mutation value considering different fitness properties between the master and the mutant flat quasispecies is given by:

$$\mu_c = 1 - \frac{f_y^{(0)} f_y^{(1)}}{f_y^{(0)} f_y^{(1)} + \sqrt{\prod_{i=0}^{1} (f_x^{(0)} - f_y^{(i)}) f_y^{(i)}}},$$

Equation (6) is recovered from the previous expression by setting $f_y^{(0)} = f_y^{(1)} = f_y$. Figure 4 displays the trans-heteroclinic bifurcation for this case. Note that the transition causes a discontinuous change in the equilibria, and the the flat quasispecies achieves population values near to 0.5 once the bifurcation takes place. The dynamics represented in the phase portraits of Fig. 4 displays the orbits in the space (x_0, y_0) flowing towards the heteroclinic connection, which can be clearly seen for the case $\mu = 0.2$. The orbits at the bifurcation value are asymptotic to the line of fixed points. When mutation rate is further increased, the heteroclinic connection vanishes (see Fig. 4c).

3. Dynamics near bifurcation threshold: transients and scaling

In this Section we focus on the dynamical behavior near the trans-heteroclinic bifurcation. It is known that transients usually become longer near bifurcation values **???**. Here we investigate the transients for the survival-of-the-flattest model given by Eqs. (5) previously discussed. The times to extinction (T_e) below and above the bifurcation are displayed in Fig. 5. Below the bifurcation the flat quasispecies becomes extinct, while above the bifurcation the flat quasispecies outcompetes the fit one. For both cases, and as μ approaches from below and above to μ_c, T_e diverges, and transients become longer and longer as $\mu \to \mu_c$. The time delay follows a power law dependence on how far the bifurcation parameter μ is below (or above) the bifurcation value μ_c. Specifically,

$$T_e \sim |\mu_c - \mu|^{-1}$$

This scaling dependence is displayed in the insets a and b of Fig. 5. The time dynamics tied to these delays are shown in the insets c and d of Fig. 5. For the case below the bifurcation, we plot two different time trajectories, using $\mu = \mu_c - 10^{-3}$ and $\mu = \mu_c - 10^{-4}$. The first case suffers a long delay, and extinction of the flat quasispecies takes plat at $t \approx 10^4$. The second case, with a slight increase of mutation rate suffers an extremely long delay, being extinction at $t \approx 7.5 \times 10^4$. Similar results are found for the time series obtained setting $\mu \gtrsim \mu_c$.

As mentioned, transient dynamics near bifurcation thresholds slow down. For instance, pitchfork bifurcations cause the so-called critical slowing down **?**. These delays for saddle-node bifurcations are named delayed transitions, and, for this particular

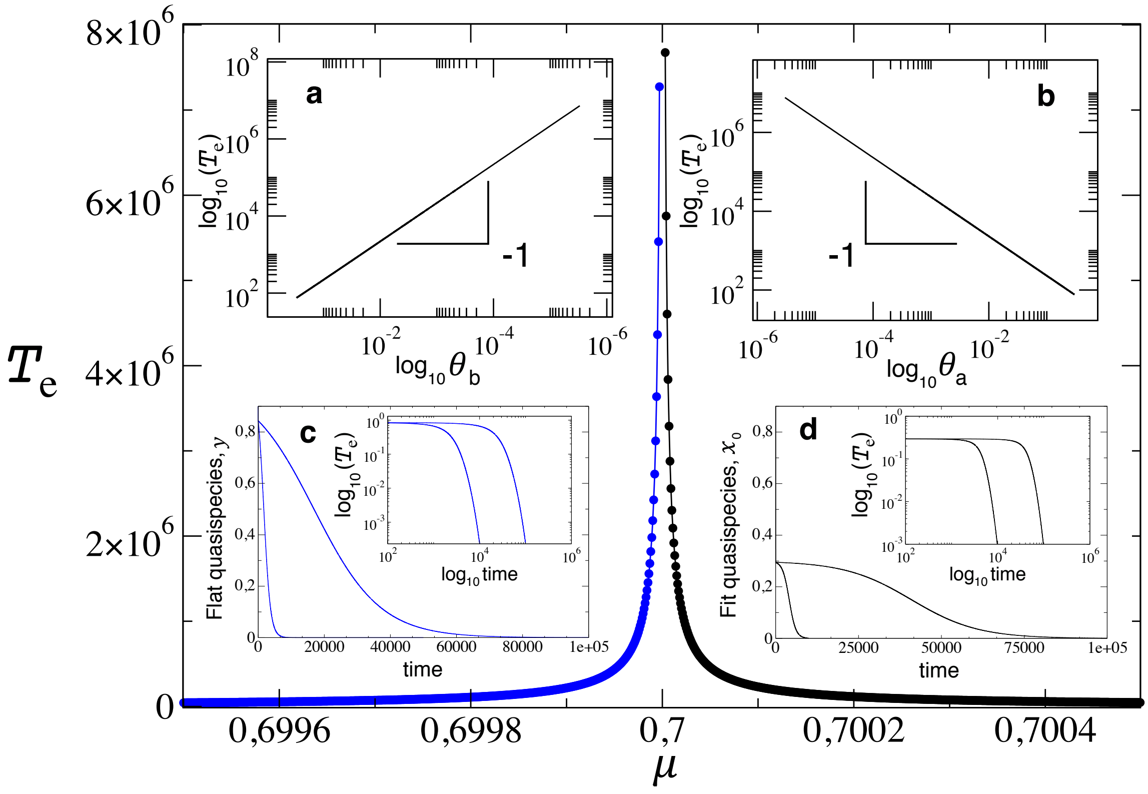

FIG. 5. **Transient behavior near a trans-heteroclinic bifurcation for the survival-of-the-flattest model.** (Main panel) Time-to-extinction (T_e) dependence on mutation rate, μ. Below μ_c the flat quasispecies, y (blue points), goes to extinction. Above μ_c the fit quasispecies (black points) becomes extinct. The upper insets display the power law dependence between the extinction time and the distance to the bifurcation value (a) below: $\theta_b = (\mu_c - \mu)^{-1}$; and (b) above $\theta_a = (\mu - \mu_c)^{-1}$ the bifurcation. The lower insets show time series in the vicinity of the bifurcation. (c) Dynamics below the bifurcation threshold with $\mu = \mu_c - 10^{-3}$ (left trajectory) and $\mu = \mu_c - 10^{-4}$ (right trajectory). Panel (d) displays the time series above bifurcation with $\mu = \mu_c + 10^{-3}$ (left trajectory) and $\mu = \mu_c + 10^{-4}$ (right trajectory). The insets of panels c and d display the same trajectories in log-log scale. In all of the panels we used $f_x^{(0)} = 1$, $f_y = 0.3$, and $\epsilon = 0$.

case, it is known that the time needed to achieve the remaining stable fixed point scales following the inverse square-root scaling law, given by $(\mu - \mu_c)^{-1/2}$ **???**. The delaying phenomena tied to the trans-heteroclinic bifurcation also involve extremely long transient in the vicinity of the bifurcation. These delays for the bifurcation studied in this manuscript involve a *trans-heteroclinic relaxation time*.

We refer the reader to the Section 2.4. in ref. **?** for the analytic derivation of this power law dependence upon bifurcation distance for the model on cancer phenotypic quasispecies discussed in the main manuscript.

M. Eigen. Selforganization of Matter and the Evolution of Biological Macromolecules. *Die Naturwissenschaften*, **58**:465, (1971).

M. Eigen, J. McCaskill, and P. Schuster. Molecular quasispecies. *J. Phys. Chem.*, **92**:6881–6891, (1988).

M. Eigen, J. McCaskill, and P. Schuster. *"The Molecular Quasi-species"*. in Advances in Chemical Physics, Volume 75 (eds. I. Prigogine and S. A. Rice), John Wiley & Sons Inc., Hoboken, NJ, USA.

A. Novick, L. Szilard. Description of the chemostat. *Science*, **112**:715–716, (1950).

J. Monod. La technique de culture continue, theorie et applications. *Ann. Inst. Pasteur*, **76**:390–410, (1950).

H. Moser. Structure and dynamics of bacterial populations maintained in the chemostat. *Cold Spring Harb Symp. Quant. Biol.*, **22**:121–137, (1957).

I.P.G. Marshall, M.F. Azizian, L. Semprini, and A.M. Spormann. Inferring community dynamics of organohalide-respiring bacteria in chemostats by covariance of *rdhA*— gene abundance. *FEMS Microbiol. Ecol.*, **87**:428–40, (2014).

R.R. Klevecz, J. Bolen, G. Forrest, and D.B. Murray. A genomewide oscillation in transcription gates DNA replication and cell cycle. *Proc. Natl. Acad. Sci. U S A.*, **101**:1200–1205, (2004).

B. Regenberg, T. Grotkjaer, O. Winther, A. Fausboll, M. Akesson, C. Bro, L.K. Hansen, S. Brunak, and J. Nielsen.. Growth-rate regulated

genes have profound impact on interpretation of transcriptome profiling in *Saccharomyces cerevisiae*. *Genome Biol.*, **7**:R107 (2006).

B.P. Tu, A. Kudlicki, M. Rowicka, and S.L. McKnight. Logic of the yeast metabolic cycle: temporal compartmentalization of cellular processes. *Science*, **310**:1152–1158, (2005).

R. Solé, J. Sardanyés, J. Díez, and A. Mas. Information catasrophe in RNA viruses through replication thresholds. *J. theor. Biol.*, **240**:353–359, (2006).

R. Solé, R. Ferrer, I. González-García, J. Quer, and E. Domingo. Red Queen dynamics, competition and critical points in a model of RNA virus quasispecies. *J. theor. Biol.*, **198**:47–59, (1999).

R. Solé, and T. Deisboeck. An error catastrophe in cancer? *J. theor. Biol.*, **228**:47–54, (2004).

R. Solé. Phase transitions in unstable cancer cell populations *Eur. Phys. J. B*, **35**:117–123, (2003).

F. M. Codoñer, J. A. Darós, R. V. Solé, and S. F. Elena. The fittest versus the flattest: experimental confirmation of the quasispecies effect with subviral pathogens. *Plos Path.*, **2**:e136, (2006).

C. O. Wilke, J. L. Wang, C. Ofria, R. E. Lenski, and C. Adami. Evolution of digital organisms at high mutation rates leads to survival of the flattest. *Nature*, **412**: 331–333, (2001).

J. Sardanyés, S. F. Elena, and R. V. Solé. Simple quasispecies models for the survival-of-the-flattest effect: The role of space. *J. Theor. Biol*, **250**:560–568, (2008).

J. Sardanyés, R. Martínez, C. Simó, and R. Solé. Abrupt transitions to tumor extinction: A phenotypic quasispecies model. *J. Math. Biol.*, **74**:1589–1609, (2017).

S. H. Strogatz, and R.M. Westervelt. Predicted power laws for delayed switching of charge-density waves. *Phys. Rev. B*, **40**:10079, (1989).

S. H. Strogatz. *"Nonlinear Dynamics and Chaos with applications to Physics, Biology, Chemistry, and Engineering"*. Westview Press, 2000.

E. Fontich and J. Sardanyés. General scaling law in the saddle-node bifurcation: a complex phase space study. *J. Phys. A: Math. Theor.*, **41**:468–482, (2008).
